# A Complete History of Ruptures and Lacerations of the Uterus, Vagina, and Perineum

**Published:** 1840-10

**Authors:** 


ART. X.

1. *Histoire Complète des Ruptures et des Déchirures de l'Uterus, du Vagin, et du Périnée.* Par F. DUPARCQUE.—*Paris,* 1836. 8vo, pp. 456.

*A Complete History of Ruptures and Lacerations of the Uterus, Vagina, and Perineum.* By F. DUPARCQUE.—*Paris.*

2. *F. Duparcque's vollstandige Geschichte der Durchlöcherungen, Einrisse, und Zereissungen des Uterus, der Vagina, und des Perineums, nebst Angabe der Praeservativen und radikalen Behandlung diesen Affectionen, &c.* Von J. F. W. NEVERMANN, M.D., &c.—*Quedlinburg und Leipzig,* 1838. 8vo, pp. xlii. und 529.

*F. Duparcque's Complete History of Perforations, Lacerations, and Ruptures of the Uterus, Vagina, and Perineum; together with the Means for the Prevention and Cure of these Affections, &c.* By J. F. W. NEVERMANN, M.D.—*Quedlinburg and Leipsic,* 1838.

M. DUPARCQUE, whose prize essay, "Traité Théorique et Pratique des Altérations Organiques de la Matrice," was noticed with commendation in the Fourth Number of this Journal, appeared shortly after, as a successful competitor for honours in the same department of medical science. In the year 1835 the Société Médicale d'Emulation of Paris offered a prize for the best essay on ruptures of the uterus and vagina, to which well-judged proceeding we owe the first complete treatise on this important subject. Since the time of Guillimeau, indeed, several instances of ruptured uterus have been recorded, and the accident has formed the theme of many monographs by English, German, or French physicians; but the cases are for the most part scattered widely through various journals, and the writers of treatises have formed their conclusions on a very limited number of observations. M. Duparcque has collected sixty-eight cases of ruptured uterus which the diligence of his German editor has increased to 230; a number whence we may make some approach to a right estimate of the circumstances enhancing the danger of the accident or which afford the greatest probability of recovery from its effects.

In his preface the author proposes to treat of the subject in four sections, considering: 1st. Ruptures of the unimpregnated uterus. 2d. Ruptures of the uterus occurring during pregnancy. 3d. Those which take place during parturition. 4th. Ruptures and lacerations of the vagina. It is much to be regretted that this simple division has not been adhered to in the body of the work, for by introducing a fifth section, on ruptures of the uterus after delivery, in which, however, lacerations of the vagina, perineum, and vulva are treated of, M. Duparcque has occasioned great and most unnecessary confusion.

After defining, in some preliminary considerations, the meaning of the terms rupture and laceration, which he regards as denoting instantaneous acts (though the phrase *chronic rupture* has been improperly employed by Madame Boivin and M. Dugés), M. Duparcque proceeds in section I. to treat of rupture of the unimpregnated uterus. This occurrence is exceedingly rare, and, indeed, impossible in the natural condition of the organ. If, however, says M. Duparcque, any cause should produce distension of the uterus, it then becomes, like the impregnated organ, exposed to the action of external injuries; or the excessive accumulation of blood, hydatids, &c. in its cavity may so attenuate its parietes as ultimately to occasion them to give way, which accident took place in the following case:

"A lady, having ceased to menstruate when forty years old, perceived at the age of fifty that a tumour was forming in her abdomen. This tumour, which was ascertained to be formed by the uterus, enlarged gradually, and ultimately attained an enormous size. Pain in the abdomen occurred at intervals, ultimately becoming insupportable, until, during a paroxysm of agony, the patient experienced a peculiar sensation in her abdomen, the pains ceased, the enlargement of the hypogastric region subsided, and she died on the following day. The cavity of the peritoneum was found filled with an enormous quantity of black blood. The uterus was dilated, its parietes generally were thick and consistent, but became thin towards the fundus, in which situation was a lacerated opening. The cervix uteri was obliterated." (pp. 13-4.)

A second similar case is recorded; but the observations of interstitial abscess of the uterus which follow are altogether irrelevant to the subject.

In section II. the author considers ruptures of the pregnant uterus, which he divides into the two classes of lacerations produced by the infliction of wounds and ruptures properly so called. Some interesting observations of recovery after wounds of the uterus are related by the author and others by the translator; but since they ought not, strictly speaking, to be classed among ruptures of the uterus, we pass to the second division of this section.

During pregnancy, the body of the uterus is the only part liable to rupture, for the cervix uteri continues to be, until the very end of gestation, the thickest part of the organ, while its situation within the pelvis defends it from external injury. The causes which most frequently produce this accident act from without, either mediately, as when violence is inflicted on the abdomen, or immediately, as when the contraction of the abdominal parietes themselves occasions the accident. The pregnant uterus, like a bladder filled with water, can yield but little to compression, and if it is subjected to force exceeding a certain degree it gives way. Ruptures thus produced take place as by *contre-coup*, that is to say, the rent takes place at a distance from the part on which the violence was inflicted, and thus it happens that the uterus usually gives way near its fundus, or a little to one side. Many circumstances, however, concur to preserve the organ from rupture, such as its extreme mobility, the defence afforded it by the abdominal muscles, the resistance of the uterine tissue, of the peritoneum investing it, and of the membranes which form the ovum. One instance indeed (Observ. vii., p. 28,) is related by the author of a blow on the abdomen, severely injuring the fœtus without at all damaging the uterine tissue. It has been doubted whether or no the placenta affords any defence from external injuries to that part of the uterus where it is inserted. M. Duparcque decides the question in the negative; but no great importance can be attached to this opinion, since he erroneously imagines the placenta to be usually adherent to the fundus of the uterus, while the observations of M. Hermann Naegele have satisfactorily proved that its ordinary situation is at the side of the organ.

Four cases are related by the author (Observs. viii.-xi., pp. 32-7,) of rupture of the peritoneal coat of the uterus, an accident to which Dr. John Clarke first called the attention of the profession. Three other cases are added by the translator (Nevermann, observs. xiv.-xvi., pp. 19-20); but two only of the seven occurred during pregnancy, the others having taken place after labour had begun. The author suggests the propriety of following Dr. C. Johnson's advice in inducing labour so soon as symptoms (which, however, are usually very obscure,) give reason to fear that this accident has taken place.

Several instances are related of rupture of the uterus from violent contraction of the abdominal muscles, and the external causes having been thus disposed of, M. Duparcque examines the influence of those which act from within the uterus. He is not disposed to allow the possibility of rupture of the uterus simply from extreme distension of its cavity. This opinion is not at all invalidated by the cases, related by M. Nevermann (p. 39), of four women, who having become pregnant after the performance of the Cæsarean section, died during utero-gestation from rupture of the womb in the situation of the former wound, since, as we shall hereafter see, the medium uniting the divided edges of the wound is often of extreme tenuity. The violence of the movements of the fœtus has been supposed capable of rupturing the womb, but from this notion M. Duparcque with reason dissents.

Next follows the consideration of those causes which predispose the pregnant uterus to rupture. M. Duparcque is of opinion that although malposition of the fœtus in utero or the presence of tumours in the neighbourhood of the womb may strongly dispose it to rupture during labour, yet the same can by no means be affirmed of them during pregnancy. Morbid conditions of the uterus, as its attenuation, softening, &c. render it very susceptible of the action of external causes, and always exist in those cases where internal causes have produced the accident. It does indeed appear that a previous degeneration of the pregnant uterus had predisposed it to rupture in every instance where the accident followed the mere contraction of the abdominal muscles or sudden movements of the body, such as may occur in convulsions or in violent fits of anger. M. Cruveilhier, considering the extremely slight causes which have produced rupture of the uterus, while the organ will bear uninjured all the exertions of a first labour, conceives that a softening of the tissue of the uterus precedes its rupture. Dr. Murphy, too, in a paper published in the year 1835 in the Dublin Journal, expressed his doubts as to the possibility of the rupture of the healthy uterus except from external violence. The remarkable case recorded by Mr. Green,* in which a cart-wheel passed over the body of a pregnant woman without injuring her uterus, and the unwarrantable experiments made some years since by Professor Ritgen, of Giessen, prove that the abdomen of pregnant women may be subjected with impunity to great compression.

Chapter II. of this section is devoted to an examination of the results of ruptures of the pregnant uterus. The accident invariably proves fatal to the fœtus, and generally, though not always, it causes the death of the mother. Sometimes the mother dies from the sudden shock to the nervous system, but her death is oftener the result of hemorrhage and of

* Medico-Chirurgical Transactions, vol. xii., part i.

the consequent effusion of blood into the abdomen. In some cases, however, where the entire ovum has escaped into the abdomen, the contraction of the uterus suppresses the hemorrhage or the retraction of the vessels, which takes place especially in a lacerated wound, prevents a copious bleeding. If the woman has survived the immediate perils of the accident, the dangers of inflammation of the uterus and peritoneum still await her and often terminate her life. Still, there are several cases on record in which the patient has ultimately recovered, and not only lived for years, but has even given birth to living children. When cases have this happy termination, the extruded fœtus becomes inclosed within a pseudo-membranous cyst, and in several instances, the cyst and its contents have undergone the calcareous transformation, and remained for twenty, twenty-five, thirty-four, and even for forty-six years in the abdomen, as was observed by Middleton, Astruc, Caldwell, Cammerarius, and others. In many cases recovery is very imperfect, and the contents of the uterus when expelled into the abdomen excite inflammation and suppuration; the bones of the fœtus are discharged through the vagina, rectum, or abdominal parietes; and the patient dies worn out by protracted suffering.

In chapter III. the symptoms are detailed; but it is only in an advanced stage of pregnancy that we can make a confident diagnosis of the nature of the accident.

In chapter IV. M. Duparcque considers the treatment of ruptures of the uterus occurring during pregnancy. He observes that the dangers to which a person is exposed by this accident depend not so much on the circumstance of the uterus being wounded as on the hemorrhage which may be produced, or on the inflammation and its consequences which may follow the escape of the fœtus into the cavity of the abdomen. If, therefore, we were called to a case where pregnancy was pretty far advanced before any very serious symptom had occurred, although the propriety of non-interference might suggest itself, yet the examples of recovery when the persons have been left to themselves are so few that the operation of gastrotomy or the vaginal Cæsarean section would probably afford a better chance of recovery. Towards the end of pregnancy, indeed, the cervix uteri is often so dilatable, that an attempt to deliver by the natural passages might well deserve a previous trial; or, if the cervix uteri were rigid, incisions might be made into it, a practice which was successfully adopted in a case that occurred to Dr. Smith, of Maidstone.* When pregnancy is not far advanced, gastrotomy is the only means of interference within our power; and M. Duparcque thinks that its employment would afford a chance of recovery to patients who are now abandoned to almost certain death. The other indications of treatment are to prevent or check the disturbance of the nervous system, to moderate or prevent hemorrhage by compression, refrigerants, &c., and, lastly, to combat inflammatory action.

Thirty observations narrated by M. Duparcque illustrate the first two sections of his book, and twenty-four additional cases in the German translation bear testimony to the diligence of M. Nevermann.

Section III. is entitled "Of ruptures of the body of the uterus during

* Communicated by Dr. Locock, in Med. Chir. Trans., vol. xiii., part ii.

labour;" but under this head M. Duparcque, with singular want of method, classes ruptures of the cervix uteri.

When rupture of the womb takes place during pregnancy that organ is purely passive; but when labour sets in the uterus is thrown into an active state, and is in consequence usually the agent of its own rupture. In the intervals of the uterine contractions, indeed, passive rupture may take place, and external causes may burst the uterus during labour as well as in the pregnant state. Ruptures of the uterus during labour differ in their causes, mechanism, and results according as the accident implicates the body or cervix of the organ; and, since a further difference exists between vertical and transverse ruptures of the cervix, M. Duparcque arranges the subject under three divisions, of which the first division treats "of ruptures of the body of the uterus during labour." The occurrence of rupture of the uterus before the membranes have given way is exceedingly rare, for the resistance afforded by the ovum to the contractions of the uterus is the same in all directions, and if the cervix does not yield, the uterus will by degrees cease to act, and will continue inert until rest has restored to it its former energy. But, although unusual, it is by no means impossible, as some have erroneously supposed: and, in confirmation of this statement, M. Duparcque relates two cases, one of which is as follows:

"A woman in labour with her sixth child, suddenly experienced violent pain, followed immediately by the escape of the liquor amnii together with a large quantity of blood, which flowed from a laceration of the lower part and right side of the womb, through which the body of the child had escaped into the cavity of the abdomen, while the head, which had driven before it the orifice of the uterus, remained in the pelvis. The limbs of the fœtus were distinctly felt through the walls of the abdomen, and the hand introduced into the uterus found that the edges of the rent were firmly contracted round the neck of the child, and that the placenta was in the abdomen. Twenty-four hours afterwards the woman died undelivered." (p. 125.)

It is after the escape of the liquor amnii that rupture of the uterus usually takes place. The contents of the womb no longer present an equal resistance in all directions: those points of the uterus in contact with projecting parts of the fœtus have to support all the force of the labour-pains. Sometimes they give way during a violent pain, or the long-continued pressure on the same part causes inflammation or gangrene, which renders the uterus liable to rupture on the slightest cause. Still, in almost all cases of rupture of the uterus, some cause or other has existed from the beginning of labour predisposing the organ to this accident. Such is attenuation of the uterine parietes, which some have imagined may be the result of natural malformation, but which more certainly follows the removal of a polypus, or a wound, or ulceration of the uterus.

Wounds of the uterus, which M. Duparcque here alludes to in passing, are among the most influential causes predisposing the organ to rupture in subsequent pregnancies or labours. Dr. Merrem, of Cologne, struck with the frequent occurrence of rupture of the uterus in women who became pregnant after having undergone the Cæsarean section, investigated the subject, and has shown* that in the nature of the cicatrix, by which

* Gemeinsame deutsche Zeitschrift für Geburtskunde, bd. iii. heft 2.

wounds of the uterus are very often closed, we have a sufficient explanation of the accident. It would appear that the wound is not unfrequently closed merely by peritoneum, or, in other cases, adhesions take place between the edges of the uterine wound and those of the divided abdominal parietes, and a fistulous opening into the womb has thus been formed, which continued open for months. Of course, in either of these cases, the uterine contractions in a subsequent labour would soon tear asunder this frail bond of union and expose the woman to all the dangers of ruptured uterus. Sometimes, indeed, the wound is united by a different process, though one not much more secure. Thus, Merrem on performing the Cæsarean section upon a woman for the second time, found the former incision four inches long and three fingers' breadth wide. It was concave instead of convex, like other parts of the walls of the uterus, and was composed of white glistening fibres, with a dilated blood-vessel running close to either margin. In the twelfth volume of the Medico-Chirurgical Transactions, Mr. Birch relates the history of a woman who recovered from a rupture of the uterus. Nine years after, having married again, she became pregnant; during labour the uterus gave way, and four days afterwards the woman died. At the post-mortem examination, at which we were present, no trace of the former rupture could be discovered; but its situation, as described by Mr. Birch, corresponded exactly to the place where the uterus had again given way. Dr. Merrem conceives that these cicatrices cause rupture by preventing the natural development of the uterus, and by interfering with its regular contractions.

Softening of the uterine tissue, irregularity of its contractions, and various alterations of its substance, such as scirrhus, (which, although they may increase the thickness of the uterine parietes, diminish its power of resistance,) may be enumerated as predisposing to this accident.

The exciting causes of rupture come next under consideration. In addition to the unskilful employment of instruments, various other causes have been assigned. M. Duparcque investigates their real influence, and illustrates by detail of cases the following conclusions: All ruptures of the uterus occurring during labour are occasioned immediately by the contractions of the organ. So long, however, as the uterus is in a healthy state, both with respect to its structure and to the manner in which it acts, these causes are insufficient to occasion rupture. The causes predisposing the uterus to rupture, act either by diminishing its organic resistance or by impairing its contractile power; and it is in this manner only that such causes as the great size or malposition of the fœtus act, unless they coincide with other predisposing causes. Even when predisposing causes exist it is usually only after the membranes have burst that rupture of the uterus occurs. Lastly, the insufficiency of the greater number of these causes, and the necessary concurrence of several, in order to produce a rupture of the uterus during labour, account for the rarity of the accident, even in those cases where its occurrence might appear to be most probable. (pp. 156-8.)

In Chapters II. and III. the results immediately following a rupture of the uterus, and the symptoms which announce the accident are detailed, without, however, any important addition to what was already known on the subject.

In the second and third divisions of this section, ruptures of the cervix uteri come under notice. The neck of the womb may be torn either vertically or in a transverse direction. The former accident usually takes place when the cervix uteri, though rigid, is susceptible of sufficient dilatation for the head to become engaged within it when the direction in which the distending force acts produces a vertical rupture. In many cases too the unskilful, or ill-judged application of instruments causes this accident. If rigidity of the os uteri should exist to so great a degree as not to admit the head of the child at all, rupture of the neck of the womb may take place in a transverse direction. Such was the accident which occurred to Mr. Scott of Norwich, when a portion of the lower segment of the uterus was torn off by the violence of the labour-pains. When, however, the tissue of the uterus generally is in a healthy state, an extreme degree of rigidity of the os uteri may exist without occasioning rupture. Thus, in a case quoted by Dr. H. Naegele,* where there existed atresia of the os uteri, the whole lower segment of the uterus, together with the child's head, were driven down to the very outlet of the pelvis: at length the accoucheur, M. Lauverjat, unable to discover the os uteri, made an incision into the organ, the child was expelled by the powers of nature, and the mother perfectly recovered.

Remarks follow on the results of ruptures of the cervix uteri, and on rupture of the periuterine region of the vagina, but want of space compels us to pass over these in order to notice some of the interesting facts collected by Dr. Nevermann in the "General Reflections," which he appends to this part of the subject. (pp. 229-45.)

It had not escaped the notice of Professor Burns or of Dr. Collins, that in a very large proportion of cases in which the uterus is ruptured, the child is of the male sex. In twenty-three of the thirty-four cases related by Dr. Collins, the children were boys; as also in fifteen of the twenty cases recorded by Dr. M'Keever; and Professor Burns's estimate that in three fourths of the cases of ruptured uterus, the children are male, probably does not exceed the truth. Dr. Collins and Dr. M'Keever relying on the measurements made by Dr. John Clarke, assign as a reason for this the greater size of the head of male children; and M. Nevermann gives us the results of the measurements of the heads of 384 children, by Professor Thulstrup, of Christiania, which fully bear out Dr. Clarke's statements.

From a comparison of thirty-three cases of ruptured uterus, Mr. Robertson, of Manchester, concludes that the accident usually occurs within thirteen hours after the commencement of labour. Dr. Collins's cases give an average of $14\frac{7}{8}$ hours for the duration of labour, and though the cases related by Dr. M'Keever do not yield exactly the same results, yet it should be borne in mind that in many of these, rupture had taken place long before the admission of the patient into the hospital. That mere protracted labour is not usually the cause of ruptured uterus is further confirmed by the fact, to which all obstetricians bear witness, of the rarity of the accident in first labours. Of the thirty-four cases recorded by Dr. Collins, seven only occurred in pri-

* Ueber die Verklebung des äussern Muttermunds als Geburtshinderniss in Medicin. Annalen. 2 bd. 2 heft.

miparæ; two only in the twenty related by Dr. M'Keever, and only one in twenty-nine cases, of which a table is given by Mr. Robertson.

Much difference of opinion has prevailed as to the part of the uterus most liable to rupture. Capuron, Boivin, Gooch, and M'Keever say, that the neck is usually the seat of laceration. Roederer, Bluff, and Ingleby state that the lower segment of the uterus is the part which, in consequence of its thinness is usually torn; and Ingleby says that laceration generally begins at the cervix, and runs in a longitudinal direction towards the body and sides of the uterus. In thirty-four cases related by Collins, the accident most frequently took place at the junction of the cervix uteri with the vagina: in thirteen at the posterior part, in twelve anteriorly, in two laterally, in one the mouth of the womb was torn, and in six the seat of the laceration was not described. In nine of the cases the peritoneum was uninjured. In no case did Dr. Collins meet with rupture of the fundus.

M. Nevermann gives the following recapitulation of the cases which he has related in his translation:

"Of 230 authenticated cases of rupture of the uterus, sixty-two occurred during pregnancy, and 168 during labour: of these latter, eighty had affected the body of the uterus, nine running in a transverse direction, and sixty-eight longitudinally, and extending backwards and to one side: in three cases, the fundus was perforated partly by the feet of the child, partly by previous ulceration, and in one case only the fundus was rent. In thirty-six cases the cervix uteri was torn longitudinally, and in fifteen in a transverse direction. In four cases the uterus was torn away from the vagina, and in twenty cases there was merely a small perforation of the body, or cervix of the uterus. In the other cases the seat of the rupture is not mentioned. The rupture does not always extend through the whole of the substance of the uterus. In four cases the peritoneum only was torn, and in five cases the uterus was torn and the peritoneum uninjured; and, in all these apparently slight cases, death ensued. The size of the ruptures and lacerations varies much; some have been seen only a quarter or half an inch in length, others nine inches long, or even involving two thirds of the uterus. Sometimes the rupture is merely a small round hole, through which fatal hemorrhage takes place; thus, in a patient of Dr. Collins, who died of sudden hemorrhage on the fifth day after delivery, a piece of the muscular substance of the uterus, about the size of a shilling, corresponding to the projection of the sacrum, was found to have given way, the peritoneal covering remaining uninjured." (pp. 233-4.)

The frequency of ruptures of the uterus is a point about which very different opinions have been entertained. Dr. Dewees thinks that they occur as often as once in every 300 labours; Burns says, one in 940; Ingleby, one in 4000; Bluff, one in 7466; M. Nevermann finds, on a comparison of 406,081 labours, that the uterus burst eighty-five times, which gives a proportion of one rupture to every 4777 labours; not one to every 4788, as M. Nevermann states.

This accident is usually regarded as one of the most fatal that can befall a female, either during pregnancy or in labour; thus, of Dr. Collins's thirty-four cases only two recovered; and of twenty cases which came under Dr. M'Keever's observation, all but two had a fatal result. M. Nevermann states, that in 106 of the 230 cases which he has detailed, the patient recovered; but surely we are not warranted in drawing any general conclusions from cases, some of which occurred in the unimpregnated uterus, others during pregnancy, others during labour.

The observations of M. Duparcque on the treatment are judicious, and well merit attention. He is an advocate for immediate delivery, which is best accomplished, when the head presents, by the application of the forceps; for attempts at turning in cases of head presentation, have in several instances ended in causing the passage of the child into the abdominal cavity. For our own parts, however, we should anticipate greater difficulty in the application of the forceps than M. Duparcque apprehends, and should think it very likely to occasion the recession of the child into the abdomen. The stethoscope will here afford us most valuable information, and enable us to choose between the forceps and the perforator; always, however, remembering, that neither is applicable unless the child is contained almost, or altogether within the uterus. If the child has passed into the abdominal cavity, an attempt should be made to deliver by turning: but if that is found impracticable or very difficult, we should have recourse to gastrotomy; and as an encouragement to its timely performance we may mention, that of thirty-four persons on whom it was performed, twenty-six, according to M. Nevermann, recovered.

The subsequent treatment presents nothing peculiar, and our limits compel us here to take leave of M. Duparcque and his translator, although the remaining chapters on ruptures of the perineum, and on vesico- and recto-vaginal fistula contain much interesting matter. M. Duparcque has done much towards filling a blank in our obstetric literature, and his book contains that which is found in no other work. Still we could have wished for a more logical arrangement of the subject, fewer unnecessary divisions or wearisome repetitions. M. Nevermann's diligence deserves all praise, and renders his book most valuable for reference, but we regret that we cannot say much of the judgment with which he has executed his task. He has added many new cases, but some of them are tedious from their length, others unsatisfactory from their brevity, and not a few have no connexion whatever with the subject. *Perpendendæ non enumerandæ sunt observationes*, is a remark (of Morgagni), which M. Nevermann would have done well to have borne in mind, and from which M. Duparcque himself might profit.

---

